# Pediatric Cirrhotic Variceal Bleed Precipitating Diabetic Ketoacidosis

**DOI:** 10.7759/cureus.11124

**Published:** 2020-10-24

**Authors:** Rachel E Bridwell, Neil P Larson, Jesse Wray, Amber Cibrario, Joshua Oliver

**Affiliations:** 1 Emergency Medicine, Brooke Army Medical Center, Fort Sam Houston, USA

**Keywords:** pediatric cirrhosis, variceal bleeding, diabetic ketacidosis

## Abstract

Cirrhosis and its associated complications such, as variceal bleeding, are rare in children, carrying significant morbidity and mortality. Leading causes of cirrhosis in the pediatric population include infection, neoplasm, and metabolic and genetic disorders, which is in contrast to the adult population. Acute gastrointestinal bleeding, as seen with variceal bleeding, has been previously associated with diabetic ketoacidosis through a multifactorial relationship. The case was complicated by hypovolemic shock whose resuscitation and subsequent transfusion was associated with cardiac overload. We highlight the need for balanced, judicious resuscitation in these individuals as well as the need for heightened awareness of coexisting pathologies such as diabetic ketoacidosis.

## Introduction

Ruptured gastroesophageal varices are a medical emergency and the most frequent source of gastrointestinal bleeding in pediatric patients with liver cirrhosis [1]. While a broad differential diagnosis exists for the etiology of underlying pediatric liver cirrhosis, a recent study cited Wilson’s disease, biliary atresia and cryptogenic sources as the most frequent culprits for patients ages 18 years of age and younger [2]. However, recent trends demonstrate an increased incidence of non-alcoholic steatohepatitis, largely reflecting the worldwide obesity problem [3,4]. Bleeding esophageal varices complicate approximately 16% of affected patients, carrying a 19% 30-day mortality [2]. There is a multifactorial relationship between acute gastrointestinal bleeding (AGIB) and diabetic ketoacidosis (DKA). Though pediatric data is limited, adult studies suggest that in cirrhotic patients hospitalized for DKA, higher presenting serum glucose levels are associated with increased rates of AGIB, and increased mortality may exist for patients experiencing AGIB compared with those who do not [5].

## Case presentation

A 17-year-old 112 kg male with a history of Acute Lymphoblastic Leukemia (ALL) in remission and Type 2 Diabetes Mellitus was brought in by ambulance to a pediatric emergency department for altered mental status. He was found unconscious in a gas station restroom with hematemesis and rectal bleeding. His mother denied a history of coagulopathy or liver dysfunction. Initial vital signs were a heart rate of 125 beats per minute, a blood pressure of 144/52 mm Hg, temperature of 97.9 ^0^F oral, a respiratory rate of 30 breaths per minute, with an oxygen saturation of 100% on 15 liters by non-rebreather mask. Physical exam was notable for mildly depressed level of consciousness though rousable, diffuse abdominal pain, and blood per rectum. Left distal femoral intraosseous access and 20 gauge intravenous forearm access were obtained en route. Initial point of care venous blood gas demonstrated a pH of 7.18, pCO^2 ^of 49.3 mm Hg, bicarbonate of 18 mmol/L, glucose of 691 mg/dL and an anion gap of 21.4 mmol/L. Relevant initial lab results included a white blood cell count of 27,600 cells/mm, hemoglobin of 9.1 g/dL, platelets of 9,000/uL, fibrinogen of 188 mg/dL (normal 200-450 mg/dL), and a D-dimer of 2.19 ug/mL (normal< 0.05 ug/mL). An insulin infusion was initiated at 0.1 units/kg/hr and one unit of packed red blood cells (pRBCs) was transfused. Due to hypotension with a repeat blood pressure of of 64/42, an epinephrine infusion was initiated at 0.05 mcg/kg/min for hypotension. After a large volume of hematemesis, the patient had a further decrease in mental status and was intubated followed by nasogastric tube placement. Simultaneously, three units pRBCs, two units fresh frozen plasma (FFP), 5 units of cryoprecipitate, 1 six-pack of platelets, and 7g of factor VII replacement were transfused and the patient was admitted to the pediatric intensive care unit (PICU) for urgent upper endoscopy by gastroenterology. On arrival in the PICU, the patient had another large bloody bowel movement with subsequent hypotension, requiring additional transfusion of two units pRBC and one unit FFP. Upper endoscopy revealed large esophageal varices with a clot in place though no further intervention was required. Emergent computed tomography revealed cirrhosis with portal hypertension. The patient’s ICU course was complicated by transfusion associated circulatory overload (TACO). However, he rapidly improved and was extubated on hospital day 3 and discharged on hospital day 8.

## Discussion

As compared to the adult population, pediatric liver dysfunction and cirrhosis occur more often secondary to infection, neoplasm, metabolic, and genetic disorders [6]. Though less common in the pediatric population, cirrhosis incurs rapid morbidity and mortality of 19% in the first 30-days following a variceal bleed [2]. In pediatric cirrhotic patients, variceal bleed is the most common cause of AGIB [7]. Decompensation from variceal bleeding, hepatic encephalopathy, and secondary bacterial infection all dramatically increase mortality in this population [8,9].

This case highlights an unusual presentation of initial identification of cirrhosis with variceal AGIB likely precipitating DKA. While DKA in a pediatric patient usually favors a hypercoagulable state, decompensated cirrhosis likely led to the coagulopathy seen in this patient; while AGIB is seen in nine percent of adult cases of DKA, very few cases of AGIB precipitating DKA in children have been reported [10]. In a retrospective case series of 25 pediatric patients with DKA associated with AGIB, one third demonstrated, esophageal erosions, Mallory-Weiss tear, or esophagitis on endoscopy [10,11]. However, there are no reported cases of DKA secondary to variceal cirrhotic bleeding. This patient had no diagnosed history of cirrhosis and thus massive AGIB was the presenting symptom of his cirrhosis. This highlights the critical and unusual nature of this case.

Pediatric variceal bleeding secondary to cirrhosis should be aggressively resuscitated using balanced transfusion of blood products as well as early prophylaxis for spontaneous bacterial peritonitis. Investigation of both concomitant coagulopathy as well as any resulting metabolic derangements should be expeditiously pursued. The patient’s severe coagulopathy again highlights this necessity. Thromboelastography guided resuscitation has demonstrated utility in the adult cirrhotic population, achieving better hemostasis with fewer blood products used [12,13]. A balanced, judicious resuscitation mitigates further complication to include TACO, as seen in this case [13]. Endoscopic variceal ligation and sclerotherapy are both used in definitive treatment of bleeding variceal in pediatric patients; ligation has been shown to reduce rates of rebleeding as compared to sclerotherapy, though the selection of therapy is multifactorial [14]. Due to the critical nature of their presentation, these patients should be admitted to the PICU with pediatric gastroenterology available for urgent endoscopy [14].

## Conclusions

Cirrhosis complicated by variceal bleeding is rare in pediatric populations but carries increased morbidity and mortality. Variceal bleeding in pediatric patients requires a balanced resuscitation with blood products and admission to a PICU with pediatric endoscopy capabilities. Emergency providers should search for concomitant derangements to include DKA in this population. To our knowledge, this is the first case where the index presentation of cirrhosis with variceal bleeding caused DKA. While rare, emergency providers should consider younger presentations of cirrhosis in comorbid patients and resuscitate aggressively but judiciously.
